# Left ventricular systolic recovery after TAVI in severe aortic stenosis: A systematic review and meta-analysis

**DOI:** 10.1016/j.ijcrp.2026.200610

**Published:** 2026-02-26

**Authors:** Elfatih A. Hasabo, Ammar Elgadi, Alaa S. Ahmed, Esraa S.A. Alfadul, Lina Hemmeda, Ruman K. Qasba, Walaa Elnaiem, Rakhtan K. Qasba, Hesham Elzomor, Ratibah Sawabi, Omar Baqal, Andreas Rück, Nawzad Saleh, Osama Soliman

**Affiliations:** a Royal College of Surgeons in Ireland (RCSI) University of Medicine and Health Sciences, Dublin, Ireland; b Cardiovascular Research Institute Dublin, and Department of Cardiology, Mater Private Network, Dublin, Ireland; c Faculty of Medicine, University of Khartoum, Khartoum, Sudan; d Sher-i-Kashmir Institute of Medical Sciences, Srinagar, India; e Green Life Medical College and Hospital, Dhaka, Bangladesh; f Department of Cardiology, University of Galway, Galway, Ireland; g Department of Cardiovascular Medicine, Mayo Clinic, Phoenix, AZ, USA; h Department of Cardiology, Karolinska University Hospital, Stockholm, Sweden; i Euro Heart Foundation, the Netherlands

**Keywords:** Aortic stenosis, Left ventricular ejection fraction, Recovery, Improvement, Transcatheter aortic valve implantation

## Abstract

**Background:**

Severe aortic stenosis (AS) imposes chronic pressure overload on the left ventricle (LV), leading to adverse remodeling and systolic dysfunction. While transcatheter aortic valve implantation (TAVI) can reverse this process, the overall prevalence and predictors of LV functional recovery are not fully understood. This study aims to assess the left ventricular ejection fraction (LVEF) recovery following TAVI.

**Methods:**

A meta-analysis was conducted in accordance with PRISMA guidelines. The PubMed, Scopus, Web of Science, and Cochrane databases were searched from inception through November 2024 for studies reporting on LVEF improvement after TAVI in patients with severe AS. The primary outcome was LVEF improvement, defined as an absolute increase of ≥10% from baseline. A random-effects model was employed for all pooled analyses to account for inter-study heterogeneity.

**Results:**

Eighteen studies encompassing 4782 patients met the inclusion criteria. In the subgroup of patients with reduced LVEF, 14 studies with a total of 3182 patients reported a LV systolic recovery of 49% (95% CI: 40-57; I2 = 97%). The recovery was observed across all follow-up periods, from immediately post-procedure to one-year. A meta-regression has identified that baseline LVEF is a predictor of LV systolic recovery (β = −0.042 (95% CI: [-0.075, −0.008], p = 0.018, I2 = 93.31%).

**Conclusions:**

TAVI is associated with substantial LV systolic recovery in nearly half of all patients with pre-existing systolic dysfunction. These findings underscore the prognostic importance of myocardial recovery in this high-risk patient population and can inform clinical decision-making and patient counseling.

## Highlights

• The true rate of LV recovery after TAVI remained unclear due to small sample sizes and inconsistent data in prior studies.
• This meta-analysis synthesized data from 4,782 patients across 18 studies to robustly quantify post-TAVI outcomes.
• Significant LV recovery was observed in 49% of patients with baseline dysfunction.
• A lower baseline ejection fraction emerged as the strongest independent predictor of myocardial recovery.
• Clinicians can now counsel high-risk patients regarding a roughly 50% probability of functional improvement post-TAVI.

## Graphical abstract

Parts of the graphical abstract were drawn using pictures from Servier Medical Art (smart.servier.com), provided by Servier, licensed under a Creative Commons Attribution 4.0 license (CC BY 4.0). (https://creativecommons.org/licenses/by/4.0/).

**Figure:**
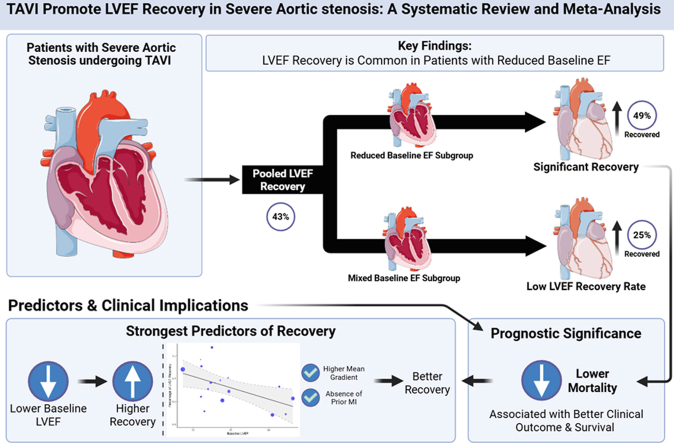

## 1 Introduction

Severe aortic stenosis is characterized by left ventricular (LV) pressure overload, leading to LV hypertrophy, concentric remodeling, and impaired LV systolic function. Many patients with symptomatic severe aortic stenosis exhibit systolic LV dysfunction. Previous studies have demonstrated that significant LV dysfunction is associated with a poor prognosis in patients undergoing surgical aortic valve replacement (SAVR) [1].

Transcatheter aortic valve implantation (TAVI) has emerged as an effective treatment for high-risk and inoperable patients with symptomatic severe aortic stenosis [2]. With growing evidence and continuous advancements in technology, TAVI is likely to become increasingly prevalent [3]. Moreover, implantation rates are projected to further increase as TAVI expands to lower-risk patient populations [4].

Following TAVI, early left ventricular ejection fraction (LVEF) improvement has been observed in approximately two-thirds of patients and is associated with better clinical outcomes [5]. Another study found that LV recovery following TAVI surpassed that of SAVR in patients with preoperative LV systolic dysfunction, with an 18-point improvement within the first week [6]. Furthermore, a study reported a significant improvement in LVEF at early follow-up after TAVI, where they found inverse correlation between hypertrophy and LVEF improvement [7].

However, a lack of LVEF improvement post-TAVI has also been reported in a substantial portion of patients with low LVEF [8]. Results from four echocardiographic studies have suggested that good LV systolic function is associated with better prognosis and lower mortality one-year post-TAVI [[9], [10], [11], [12]].

Most studies that investigated LV recovery after TAVI have included relatively small participant numbers. Moreover, limited information exists regarding LVEF improvement and recovery in patients who underwent TAVI. This systematic review aims to consolidate and synthesize existing data to assess LV systolic recovery and its associated predictors following TAVI.

## 2 Methods and materials

### 2.1 Protocol and reporting standards

This systematic review and meta-analysis aimed to determine the proportion of patients achieving LV systolic recovery following TAVI and identify predictors of this recovery in patients with aortic stenosis. It was designed and executed in strict accordance with the Cochrane Handbook for Systematic Reviews of Interventions [13] and was The findings are reported according to the Preferred Reporting Items for Systematic Reviews and Meta-Analyses (PRISMA) 2020 statement to ensure transparency and completeness [14].

### 2.2 Search strategy

A comprehensive and systematic literature search was conducted across four major electronic databases: PubMed, Scopus, Web of Science, and the Cochrane Library. The search was performed to include all studies from database inception to November 2024. The search strategy combined keywords and medical subject headings related to TAVI: (“Transcatheter Aortic Valve Implantation” OR TAVI OR “Transcatheter aortic valve replacement” OR TAVR OR “Percutaneous aortic valve replacement” OR PAVR OR “transcatheter heart valve” OR THV) AND (Recovery OR Recovered OR Remodeling OR “Change in EF” OR “Change in Ejection Fraction” OR “Change in left ventricular ejection function” OR “Change in Left Ventricular Ejection Fraction” OR “Left Ventricular Ejection Fraction Improvement” OR “global longitudinal strain” OR GLM OR “Left ventricular mass index” OR LVMi OR “Left ventricular global longitudinal strain” OR LV-GLS OR “ventricular mass index” OR normalization OR normalization). The detailed search strategy for each database is provided in Supplementary Table 1.

### 2.3 Eligibility criteria

Studies were included if they met the following criteria: were randomized controlled trials (RCTs), cohort studies, or case-control studies; involved patients with severe aortic stenosis undergoing TAVI; reported the proportion or number of participants who achieved LV systolic recovery, as defined by the individual study; and were published in English. Studies were excluded if they were abstracts, review articles, opinion papers, study protocols, or in vitro/animal studies.

#### 2.3.1 Study selection

The study selection process was performed in two stages. First stage, E.A.H. conducted the searches in databases and removed duplicate studies. In the second stage, multiple independent reviewers (S.S.A., A.E., A.S.A., E.S.A.A., L.H., R.K.Q., W.E., R.K.H., H.E., R.W.) screened the titles and abstracts of all identified records. Subsequently, the full texts of potentially relevant articles were retrieved and assessed for final eligibility. Any disagreements or discrepancies during the screening process were resolved through discussion and consensus with a senior author (E.A.H.).

#### 2.3.2 Data extraction

Data were extracted from the included studies by two independent reviewers using a standardized data extraction form. The extracted information included study characteristics (first author, publication year, country, study design), baseline patient characteristics (sample size, age, sex, comorbidities, surgical risk scores), procedural details (TAVI device type), and factors associated with LV systolic recovery following TAVI. The primary outcome of interest was the prevalence of LV systolic recovery.

#### 2.3.3 Quality assessment

The methodological quality and risk of bias of the included studies were independently assessed by two reviewers. For observational cohort and case-control studies, the Newcastle-Ottawa Scale (NOS) was used [15]. This tool assesses studies based on three domains: selection of study groups, comparability of groups, and ascertainment of the outcome or exposure.

For RCTs, the Cochrane Risk of Bias tool (version 5.1.0) was used to assess the quality of clinical trials. It evaluates potential biases across several domains, including random sequence generation, allocation concealment, blinding, incomplete outcome data, and selective reporting [13]. The detailed results of these quality assessments are presented in Supplementary Table 2 and Supplementary Table 3.

### 2.4 Data analysis

The meta-analysis was performed using R software (version 4.4.2). Given the anticipated clinical and methodological diversity among the included studies (e.g., differences in patient populations, follow-up durations, and specific TAVI technologies), a random-effects model was chosen a priori for all pooled analyses. The pooled prevalence of LVEF improvement and its corresponding 95% confidence interval (CI) were calculated using the “metaprop” function. Statistical heterogeneity across studies was quantified using the I^2^ statistic, with values of 25%, 50%, and 75% considered to represent low, moderate, and high heterogeneity, respectively. A chi-square test for heterogeneity was also performed, with a p-value <0.10 indicating significant heterogeneity. To investigate sources of heterogeneity and explore the robustness of the findings, several pre-specified subgroup analyses were conducted based on: baseline LVEF status (studies including only patients with reduced EF versus those with a mixed population of reduced and preserved EF), duration of follow-up post-TAVI, type of TAVI device used (balloon-expandable vs. self-expandable), and era of publication (before vs. after 2018). A meta-regression was used to find the effect of baseline LVEF or publication year on LV systolic recovery.

## 3 Results

### 3.1 Literature search

**Fig. 1:**
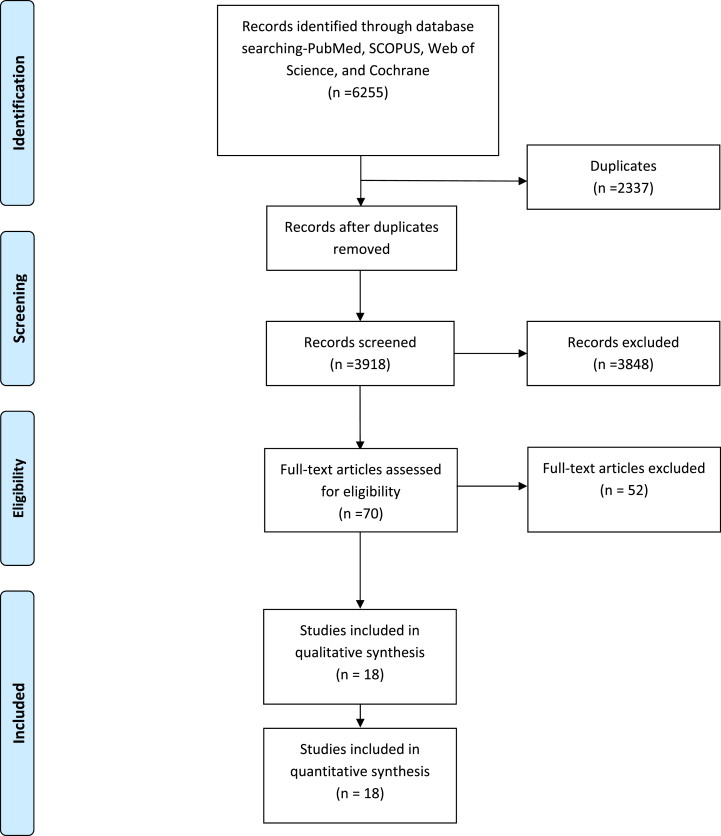
PRISMA flow diagram of study selection.

The initial search yielded 6126 studies across PubMed, Scopus, Web of Science, and the Cochrane Database. After removing duplicates, 3731 studies were screened by title and abstract, and 441 underwent full-text screening. Eighteen studies were ultimately deemed eligible for inclusion in the qualitative and quantitative synthesis. The PRISMA flow diagram in Fig. 1 details the study selection process. The final analysis included 18 studies, which collectively enrolled a total of 4782 patients with severe aortic stenosis who underwent TAVI, consist of the following: eight studies were prospective cohort studies [1,7,[16], [17], [18], [19], [20], [21]], seven were retrospective studies [6,[8], [22], [23], [24], [25], [26]], and three were clinical trials [2,5,27].

#### 3.1.1 Risk of bias

The quality assessment revealed that the 15 included observational studies were generally of high quality, with NOS scores ranging from 5 to 6 out of a maximum of 6 points. The three included RCTs were judged to have a high risk of performance and detection bias due to their unblinded, open-label design, which is inherent to procedural trials of this nature. However, the risk of attrition and reporting bias was low in these trials (Supplementary Table 2 and Supplementary Table 3).

### 3.2 Device type and follow-up

The included studies utilized a range of TAVI devices, including both balloon-expandable valves (e.g., Sapien series; Edwards Lifesciences, Irvine, CA, USA) and self-expandable valves (e.g., Medtronic CoreValve/Evolut series; Medtronic, Minneapolis, MN, USA). The follow-up period for assessing LV systolic recovery varied widely, ranging from immediately post-procedure to one year. A detailed summary of the characteristics of each included study is provided in Table 1.

**Table 1:** Summary characteristics of included studies.

Study	Study design	Total	Definition of LV systolic recovery	Baseline ejection fraction (EF) status	LV systolic recovery at the end of follow-up, %	Type of device used in TAVI	Duration of follow-up	Vascular access		
								Transfemoral	Transapical	Direct aortic
Elmariah et al., 2013 [27]	Clinical trial	108	≥10% LVEF improvement at 30 days.	Reduced EF	48 (51.6%)	Balloon-expandable valve (SAPIEN)	30 days	-	-	-
Bauer et al., 2013 [6]	Retrospective study	31	≥10% LVEF improvement	Reduced EF	21 (68%)	Balloon-expandable valve (Edwards-Sapien)	7 days	22 (71%)	9 (29%)	-
Barbash et al., 2014 [16]	Prospective study (post hoc)	99	10% Improvement in LVEF	Reduced EF	24 (24.2%)	Balloon-expandable valve (SAPIEN), Self-expandable valve (CoreValve)	30 days	69 (69.7%)	30 (30.3%)	0 (0%)
Passeri et al., 2015 [2]	Clinical trial	46	≥10% LVEF improvement at 30 days.	Reduced EF	22 (48.7%)	Balloon-expandable valve (SAPIEN)	30 days	46 (100%)	-	-
Chen et al., 2016 [22]	Retrospective study	60	≥10% LVEF improvement post-TAVI	Reduced EF	30 (50%)	Balloon-expandable valve (SAPIEN, SAPIEN XT)	Post TAVI	-	-	-
Dauerman et al., 2016 [5]	Clinical trial	156	≥10% LVEF improvement at 30 days.	Reduced EF	97 (62.2%)	Self-expandable valve (CoreValve)	30 days	116 (74.4%)	non transfemoral 40 (25.6%)	
Angelillis et al., 2017 [23]	Retrospective study	252	≥10% LVEF improvement	Reduced EF	121 (48%)	Self-expandable valve (CoreValve, Evolut R)	30 days	200 (79.4%)	-	14 (5.6%)
D'Onofrio et al., 2017 [17]	Prospective study	122	5% Improvement in LVEF	Mixture of reduced and preserved EF	27 (22.1 %)	Balloon-expandable valve (SAPIEN, SAPIEN XT, SAPIEN 3)	Post TAVI	-	122 (100%)	-
Deste et al., 2018 [7]	Prospective study	46	Increase in LVEF at one month	Reduced EF	24 (52.2%)	Balloon-expandable valve (SAPIEN XT), self-expandable valve (CoreValve, Accurate neo TF Symetis)	30 days	46 (100%)	-	-
Komatsu et al., 2020 [24]	Retrospective study	99	≥10% LVEF improvement at 1 month	Reduced EF	42 (42.4%)	Balloon-expandable valve (SAPIEN, SAPIEN XT, SAPIEN 3), Self-expandable valve (CoreValve Classic, Evolut R, Evolut PRO)	30 days	92 (92.9%)	-	-
Han et al., 2021 [8]	Retrospective study	109	≥10% LVEF improvement within 6 months of the procedure.	Reduced EF	39 (36%)	Balloon-expandable valve (SAPIEN), self-expandable valve (Evolut, CoreValve)	6 months	-	-	-
Jeong et al., 2021[1]	Prospective study	694	Immediate improvement: ≥5% increase in EF immediately after TAVI	Mixture of reduced and preserved EF	160 (23.1%)	Balloon-expandable valve (SAPIEN XT, SAPIEN 3), self-expandable valve (CoreValve, Evolut R, Evolut Pro), mechanically-expandable valve (Lotus)	Post TAVI	-	-	-
Kolte et al., 2022 [18]	Prospective study	659	≥10% LVEF improvement at 30 days.	Reduced EF	216 (32.8%)	Balloon-expandable valve (SAPIEN, SAPIEN XT and SAPIEN 3)	30 days	659 (100%)	-	-
Kuneman et al., 2022 [25]	Retrospective study	560	≥10% LVEF improvement & ≥5% LVEF improvement	Mixture of reduced and preserved EF	≥10% LVEF improvement 121 (22%) ≥5% LVEF improvement 233 (42%)	Balloon-expandable valve (SAPIEN, SAPIEN XT, SAPIEN 3), Self-expandable valve (CoreValve, Evolut R, Evolut Pro)	6 months	560 (100%)	-	-
Wilde et al., 2023 [26]	Retrospective study	219	Left ventricular reverse remodeling: ≥10% increase in LVEF and ≤15% decrease in LVESV.	Reduced EF	169 (77.2%)	Self-expandable valve, Balloon-expandable valve	Median 5.2 months (IQR, 2.7 -8.1 months),	200 (91.3%)	-	-
Bernhard et al., 2024 [19]	Prospective study	608	5% Improvement in LVEF or 5% decrease in LVEDD at 12 months compared to baseline	Mixture of reduced and preserved EF	209/428 (48.8 %)	Balloon-expandable valve, self-expandable valve, mechanically-expandable valve	12 months	593 (98.3%)	-	-
Witberg et al., 2024 [20]	Prospective study	914	≥10% LVEF improvement within 60 days after TAVI	Reduced EF	544 (59.5%)	-	Within 60 days	841(92.3%)	-	-
Reichl et al., 2024 [21]	Prospective study	384	≥10% LVEF improvement within 30 days after TAVI	Reduced EF	155 (40.4%)	-	30 days	-	-	-

Data are presented as number (percentage).

Abbreviations: EF: Ejection fraction, LVEDD: Left ventricular end diastolic dimension, LVEF: Left ventricle ejection fraction, LVESV: Left ventricular end systolic volume, TAVI: Transcatheter aortic valve intervention.

### 3.3 Patient characteristics

The patient populations were predominantly elderly, with mean ages ranging from 79.7 to 85 years. Passeri et al. (2015)(2) reported the highest mean age (85 ± 8 years), while Jeong et al. (2021)(1) reported the lowest (79.7 ± 5.4 years). Jeong et al. (2021)(1) and Witberg et al. (2024)(20) included relatively younger populations compared to other studies.

Most studies included a majority of male participants, with the exception of D'Onofrio et al. (2017)(17), where males comprised approximately half of the cohort. Han et al. (2021)(8) had the highest proportion of males (76.2%), followed by Komatsu et al. (2020)(24).

The Society of Thoracic Surgeons (STS) score and logistic EuroScore II varied across studies. These scores were categorized as high (STS >8), intermediate (STS 4-8), and low risk (STS <4) [28].

All studies included patients with severe aortic stenosis undergoing TAVI via self and ballon-expandable valves, with the exception of Jeong et al. (2021) [1] and Bernhard et al. (2024)(19), which included a subset of patients undergoing implantation of mechanically-expandable valves. Further details were provided in Table 1, Table 2.

**Table 2:** Baseline clinical and echocardiographic characteristics of the study population.

Study	Age, years	Male	Logistic EuroSCORE	STS score	Baseline EF	eGFR (ml/min)	Risk factors, number (%)									NYHA class, number (%)			
							HTN	Dyslipidemia	DM	Smoker	CAD	Previous MI	Previous revascularization	PCI	CABG	1	2	3	4
Elmariah et al., 2013 [27]	83 ± 7	74 (68.5%)	-	12.2 ± 3.7	37.1 ± 9.2		-	-	-	-	87 (80.6%)	45 (41.7%)	-	41 (38.3%)	92 (41.1%)	-	4 (3.7%)	104 (96.3%)	
Bauer et al., 2013 [6]	83 ± 6	21 (67.7%)	-	-	32 ± 9	-	-	-	-	-	-	5 (16.1%)	0 (0%)	-	-	-	-	-	-
Barbash et al., 2014 [16]	84 ± 7	63 (64%)	38 ± 27	12 ± 5	33 ± 8	-	91 (93%)	-	34 (35%)	19 (30%)	68 (87%)	28 (30%)	-	32 (34%)	48 (50%)	-	-	-	-
Passeri et al., 2015 [2]	85 ± 8	24 (52.2%)	-	13.2 ± 6.4	35.8 ± 8.9	-	-	-	-	-	34 (73.9%)	12 (26.7%)	-	14 (30.4%)	21 (45.7%)	-	2 (4.3%)	44 (95.7%)	-
Chen et al., 2016 [22]	-	39 (60%)	-	-	-	-	-	-	-	-	51 (60%)	24 (40%)	-	20 (33.3%)	28 (46.6%)	-	-	23 (38.33%)	37(61.66%)
Dauerman et al., 2016 [5]	82.5 ± 9.1	109 (69.9%)	-	-	-		139 (56.1%)	134 (87.5%)	66 (42.3%)	93 (59.6%)	139 (89.1%)	78 (49.9%)	-	63 (40.4%)	86 (55.12%)	103 (66%)			53 (34%)
Angelillis et al., 2017 [23]	80.9 ± 8.1	130 (51.6%)	22.9 (15–35)	7.13 ± 5.48	34.7 ± 7.4	GFR <30 mL/min: 78 (31%)	197 (78.2%)	-	65 (25.7%)	-	72 (28.5%)	-	-	57 (22.6%)	-	-	-	199 (79%)	
D'Onofrio et al., 2017 [17]	80.1 ± 6.1	61 (50)	20.9 ± 12.1	8.2 ± 7.9	54.5 ± 12	-	114 (93.4%)	-	35 (28.7%)	-	-	31 (25.4)	-	-	-	-	-	88 (72.2%)	-
Deste et al., 2018 [7]	80 ± 3	27 (58.6%)	13.9 ± 7	5 ± 1.7	-	-	43 (93.4%)	-	15 (32.6%)	-	-	10 (21.7%)	-	-	-	-	-	38 (82.6%)	
Komatsu et al., 2020 [24]	81.7 ± 8.5	73 (73.7%)	-	8.1 ± 4.3	33.3 ± 12.6	-	89 (89.9%)	-	37 (37.4%)	-	-	40 (40.4%)	-	33 (33.3%)	24 (24.2%)	-	-	-	-
Han et al., 2021 [8]	80 ± 9.7	83 (76.2%)	-	-	32.2 ± 10.1	-	78 (71.6%)	-	39 (35.8%)	-	-	20 (18.4%)	39 (35.8%)	21 (19.3%)	25 (22.9%)	-	-	-	-
Jeong et al., 2021(1)	79.7 ± 5.4	353(50.9%)	12.7 ± 11	3.9 ± 2.9	63 ± 25.8	-	610 (87.9%)	527 (75.9%)	346(49.9%)	84(12%)	-	31(4.5%)	-	196(28.2%)	33(4.8%)	-	-	-	-
Kolte et al., 2022 [18]	82.6 ± 7.2	468 (71%)	-	8.5 ± 4.2	37.2 ± 8.4	-	598 (90.7%)	-	245 (37.2%)	-	529 (80.3%)	200 (30.3%)	-	236 (35.8%)	251 (38.1%)	-	-	-	-
Kuneman et al., 2022 [25]	80 ± 7	296 (53%)	-	-	51 ± 11	61 ± 20	407 (73%)	338 (61%)	159 (28%)	76 (14%)	351 (63%)	112 (20%)	293 (52%)	173 (31%)	120 (21%)	231 (41%)		327 (59%)	
Wilde et al., 2023 [26]	80 ± 6	141 (64.4%)	33.8 ± 17.6	8.0 ± 6.3	35.0 ± 10.0	49.4 ± 20.7	-	-	70 (32.0)	-	167 (76.3)	65 (30.0)	-	103 (47.3)	-	-	-	-	40 (18.3%)
Bernhard et al., 2024 [19]	81.1 ± 6.6	337 (55.4%)	-	3.2 (2.0–5.9)	-	55 (39–72)	554 (91.1%)	424 (69.7%)	181 (29.8%)	181 (29.8%)	285 (46.9%)	71 (11.7 %)	-	-	-	19 (3.1 %)	273 (44.9%)	272 (44.7%)	44 (7.2 %)
Witberg et al., 2024 [20]	79.8 ± 7.8	603 (66%)	-	7.2 ± 1.4	27.3 ± 5.7	53.1 ± 25.6	702(76.4%)	-	328(35.4%)	-	-	265(28.9%)	-	323(35.4%)	163(17.8%)	-	-	-	-
Reichl et al., 2024 [21]	-	-	-	-	-	-	-	-	-	-	-	-	-	-	-	-	-	-	-

Data are presented as mean (SD), number (percentage), median (IQR).

Abbreviation: CABG: Coronary artery bypass grafting, CAD: Coronary artery disease, DM: Diabetes mellitus, EF: Ejection fraction, eGFR: Estimated glomerular filtration rate, GFR: Glomerular filtration rate, HTN: Hypertension, IQR: Interquartile range, MI: Myocardial infarction, NYHA: New York Heart Association, PCI: Precutaneous coronary intervention, SD: Standard deviation, STS score: Society of thoracic surgeons score.

### 3.4 Baseline characteristics in LVEF recovered vs. non-recovered groups

Eleven studies stratified baseline characteristics into recovered and non-recovered groups based on LVEF improvement [1,5,[16], [17], [18],[8], [20], [21], [22], [23], [24],^26^,^29^]. Baseline characteristics were generally similar between the two groups in most studies. However, a history of myocardial infarction (MI), percutaneous coronary intervention (PCI), and diabetes mellitus were more prevalent in the non-recovered groups in many studies, although these differences were not always consistent (Supplementary Table 4).

Furthermore, recovered groups tended to have lower baseline EF compared to non-recovered groups. An exception was observed in Witberg et al. [20], where baseline EF was comparable across groups with no EF improvement, EF improvement, and EF normalization. Further details regarding baseline characteristics stratified by LV systolic recovery status are provided in Supplementary Table 4.

### 3.5 Prevalence of LV systolic recovery and its associated factors

The definition of LV systolic recovery varied across studies. Most studies defined improvement as an increase in LVEF of ≥10%, with exceptions in D'Onofrio et al. [17], Jeong et al. [1], and Bernhard et al. [19], which used a 5% increase threshold. Kuneman et al. [25] reported EF improvement for both 5% and 10% thresholds. The definition used by each study was detailed in Table 1.

**Fig. 2:**
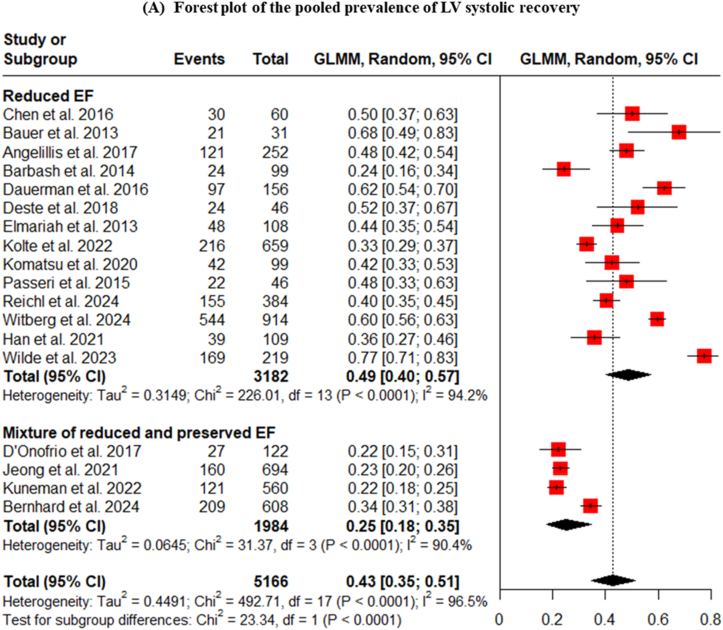

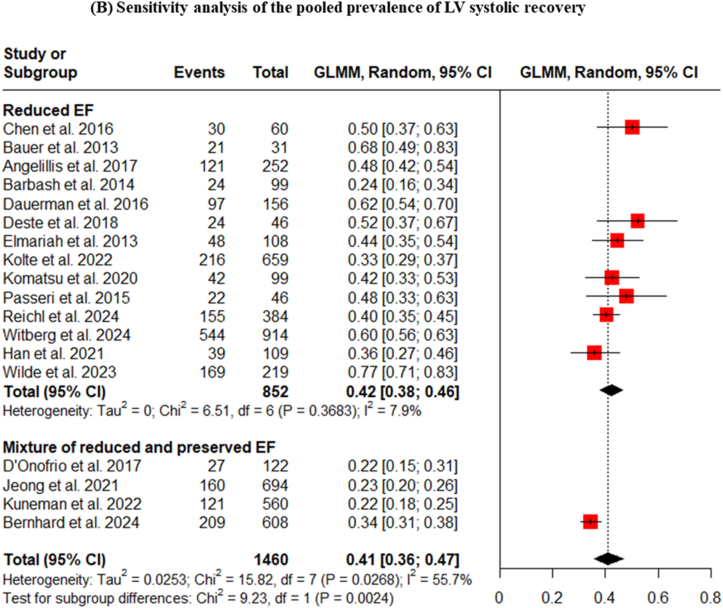
Pooled prevalence and sensitivity analysis of LV systolic recovery post-TAVI.

The primary meta-analysis, including all 18 studies, found that the pooled prevalence of LVEF improvement following TAVI was 43% (95% CI: 35-51). As anticipated, there was a very high degree of statistical heterogeneity among the studies (I^2^ = 97%, p < 0.0001) (Fig. 2A).

A critical pre-specified subgroup analysis was conducted based on the baseline LVEF characteristics of the study populations. This analysis revealed a stark and clinically important difference. In studies that exclusively enrolled patients with reduced baseline LVEF, the pooled prevalence of recovery was 49% (95% CI: 40-57). In contrast, for studies that included a mixed population of patients with both reduced and preserved LVEF, the pooled prevalence of improvement was only 25% (Fig. 2A). This demonstrates that the potential for significant LV systolic recovery is predominantly concentrated in the patient cohort with pre-existing systolic dysfunction. A sensitivity analysis performed on the reduced EF group showed a pooled prevalence of 42% with substantially reduced heterogeneity (I^2^ = 5.9%) (Fig. 2B).

### 3.6 Subgroup and sensitivity analyses

1. **Temporal Pattern of LV systolic recovery according to follow-up time and baseline EF**

**Fig. 3:**
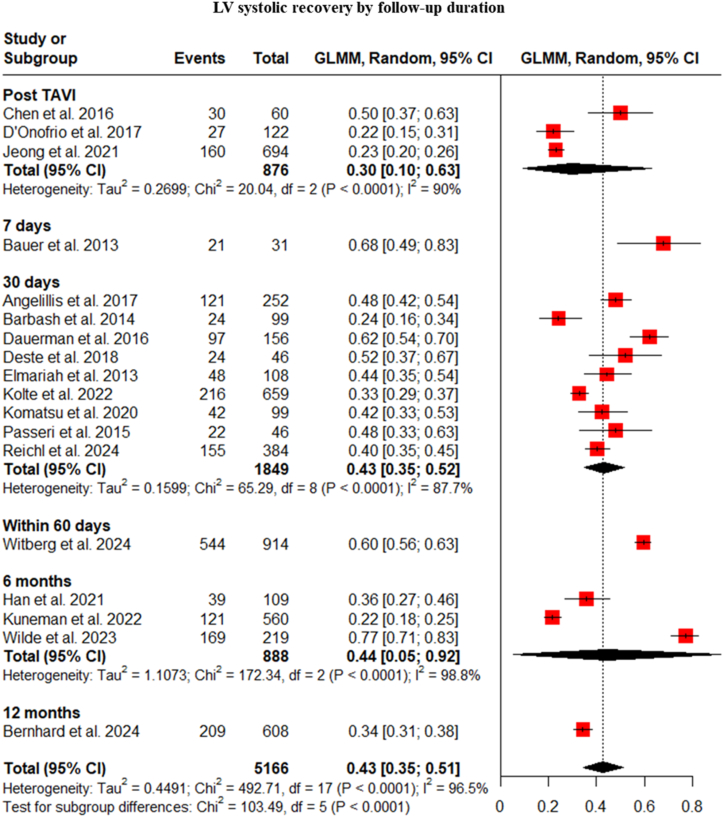
Subgroup analysis of LV systolic recovery by follow-up duration.

The timing of LV systolic recovery was assessed at various follow-up points. The pooled analysis showed recovery rates of 30% immediately post-TAVI, 68% at 7 days, 43% at 30 days, 60% within 60 days, 45% at 6 months, and 34% at 12 months. The pooled LV systolic recovery rates at these time points were (Fig. 3). At 7 days post-TAVI, we must be aware that the 68% recovery is derived from a single small study (Bauer et al., 2013, n = 31) [6], which cannot be generalized to infer early LV systolic recovery.

**Fig. 4:**
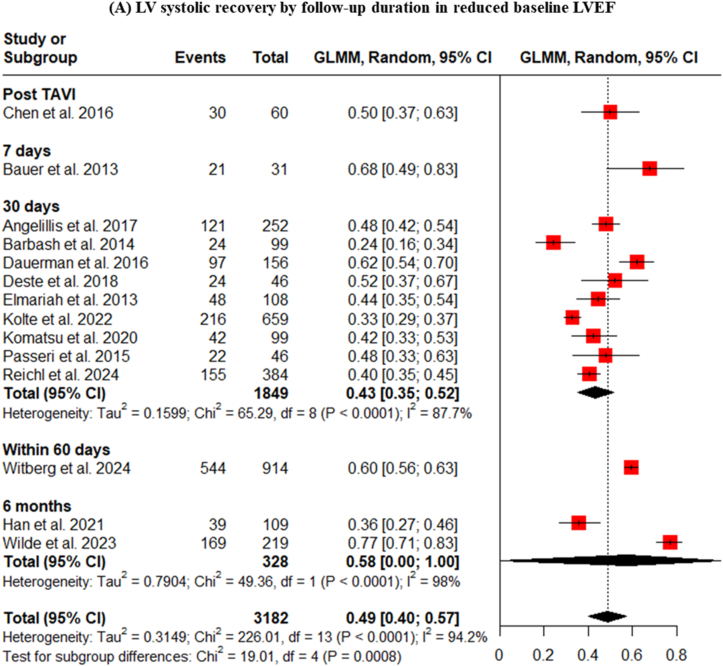

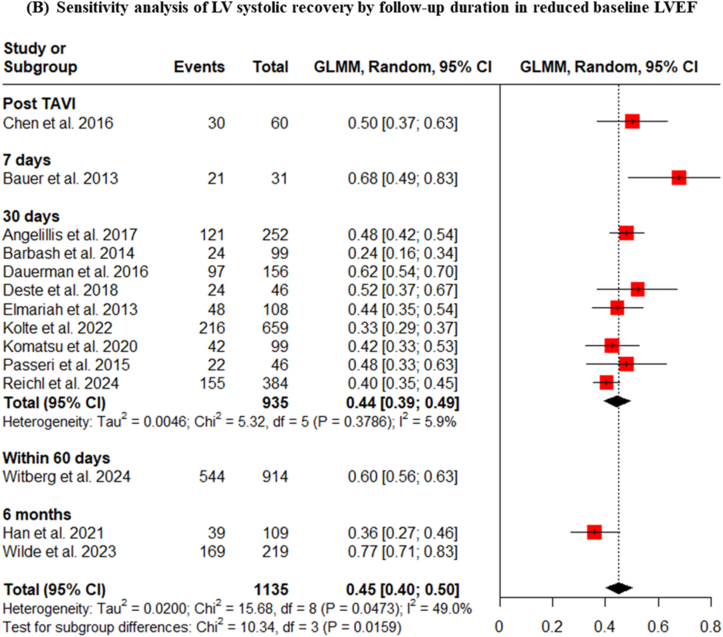
LV systolic recovery in patients with reduced baseline LVEF, stratified by follow-up duration.

When focusing specifically on the more relevant subgroup of patients with reduced baseline EF, a similar dynamic pattern was observed, with pooled recovery rates of 50% immediately post-TAVI, 68% at 7 days, 43% at 30 days, 60% within 60 days, and 58% at 6 months (Fig. 4A). These findings suggest that a substantial portion of the myocardial recovery occurs very early after afterload reduction, often within the first week. Sensitivity analysis led to decrease the heterogeneity for LVEF improvement within 30 days after TAVI and showed 44% (I2 = 5.9%, p = 0.3786) (Fig. 4B).

2. **Influence of Device Type on LV Systolic Recovery**

**Fig. 5:**
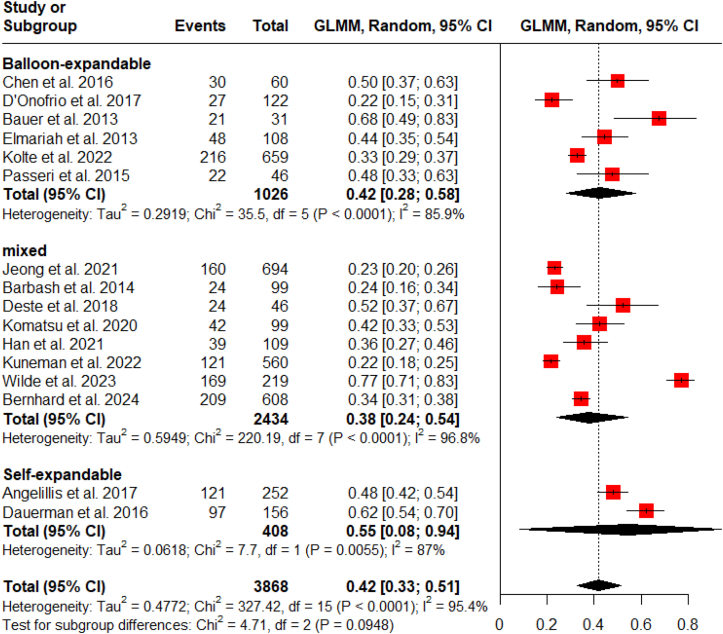
Subgroup analysis of LV systolic recovery by TAVI device type.

Subgroup analyses were performed to determine if LV systolic recovery was influenced by the type of valve implanted or the era of the procedure. The analysis showed no statistically significant difference in recovery rates between patients receiving balloon-expandable valves (42%), self-expandable valves (55%), or a mixture of both types (38%) (Fig. 5).

3. **Influence of era of TAVI implantation on LV systolic improvement**

**Fig. 6:**
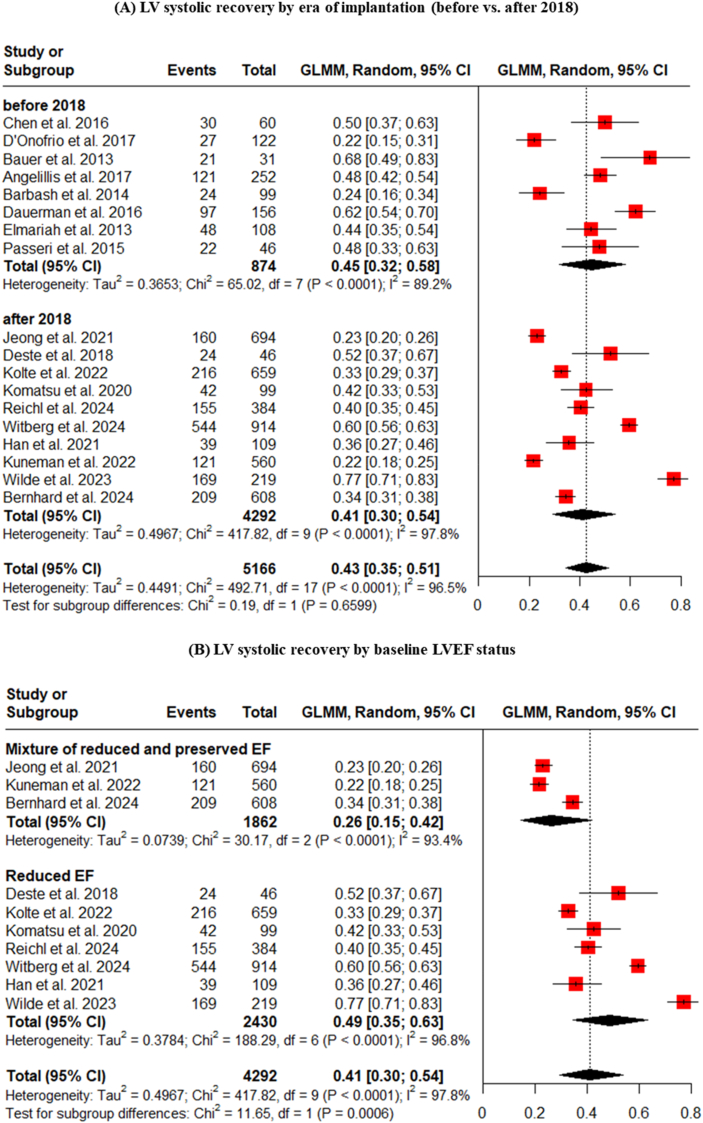

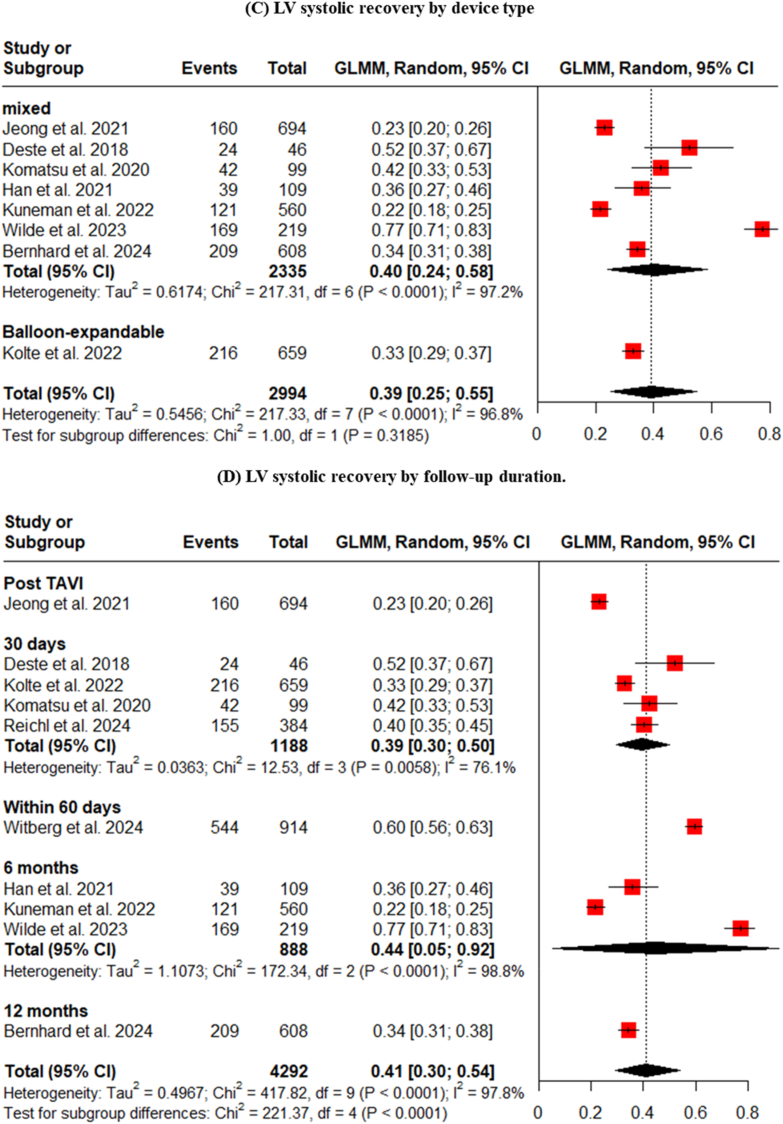
Subgroup analyses of LV systolic recovery by (A) Era of implantation (before vs. after 2018), (B) Baseline LVEF status, (C) Device type, and (D) Follow-up duration.

Even within the modern era (post-2018), the pooled percentage of LVEF improvement was 41% (vs. 45% for studies before 2018) (Fig. 6A), and the most powerful determinant of recovery remained the baseline LV function. The analysis of studies published after 2018 confirmed the dominant signal seen in the overall cohort: patients with reduced EF had a 49% rate of recovery, compared to just 26% in mixed-EF populations (Fig. 6B). This finding reinforces that while TAVI technology and techniques have evolved, the fundamental biological substrate of the myocardium is the primary driver of functional recovery.

Studies after 2018 didn't show any difference when compared with studies before 2018 or when categorized by device type or follow-up duration (Fig. 6).

### 3.7 Predictors of LV systolic recovery

Eleven of the included studies provided data on factors associated with LV systolic recovery, often comparing baseline characteristics between patients who did and didn't recover (Supplementary Table 5). Furthermore, several studies performed univariate and multivariate logistic regression analyses to identify independent predictors of recovery (Table 3 and Supplementary Table 5). Across these analyses, the most consistent and statistically significant predictor of LV recovery was a lower baseline LVEF. Other factors identified in some studies included the absence of significant coronary artery disease or prior myocardial infarction, and a higher baseline mean aortic valve gradient (Table 3 and Supplementary Table 5).

**Table 3:** Pooled predictors of LV systolic recovery following TAVI.

Factor	Association with LV systolic Recovery	Supporting Studies (Multivariate Analysis)
Lower Baseline LVEF	Strong Positive Predictor	Kuneman et al. (2022), Kolte et al. (2022), Bernhard et al. (2024), Elmariah et al. (2013)
Higher Baseline Mean Gradient	Positive Predictor	Dauerman et al. (2016), Witberg et al. (2024)
Absence of Prior Myocardial Infarction	Positive Predictor	Dauerman et al. (2016), Witberg et al. (2024)
Absence of Coronary Artery Disease	Positive Predictor	Kuneman et al. (2022) (Univariate)
Absence of Prior PCI	Positive Predictor	Kuneman et al. (2022) (Univariate)
Absence of Hypertension	Positive Predictor	Kuneman et al. (2022) (Univariate)
Good Renal Function (eGFR >60)	Positive Predictor	Witberg et al. (2024)

**Fig. 7:**
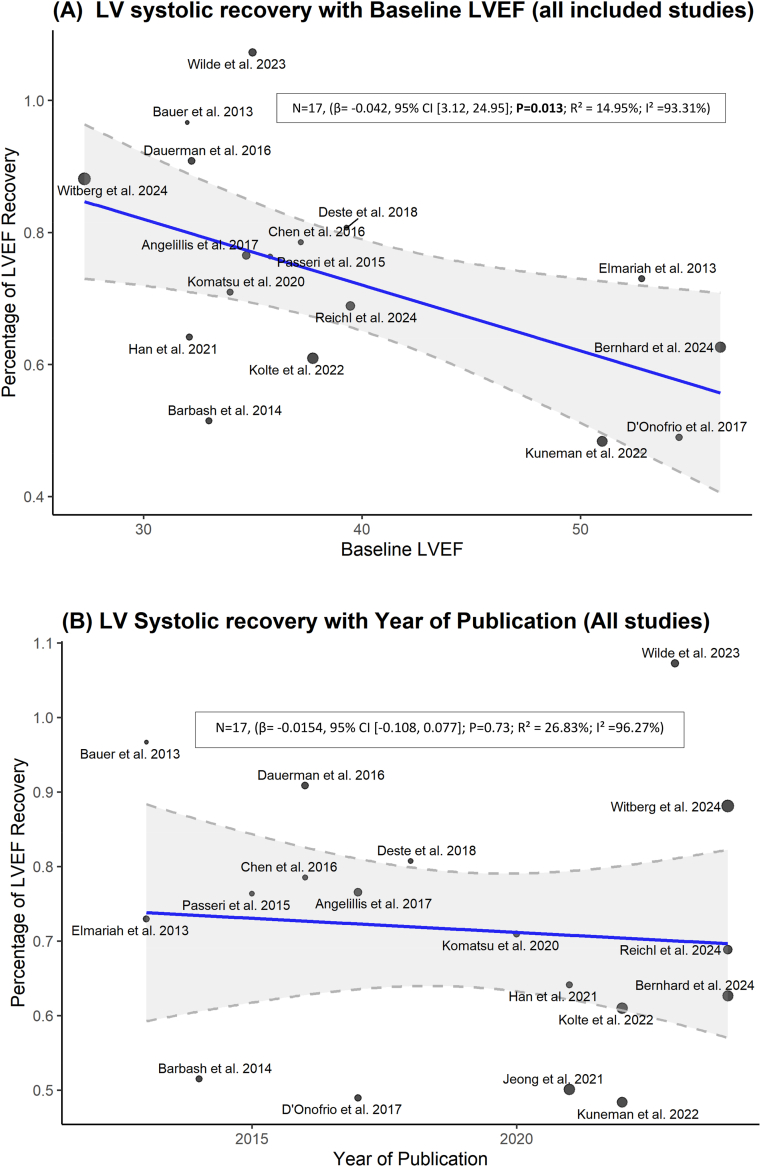
Meta-regression of LV systolic recovery (all studies with both ≥10% and ≥5% thresholds) against (A) baseline LVEF and (B) publication year.

**Fig. 8:**
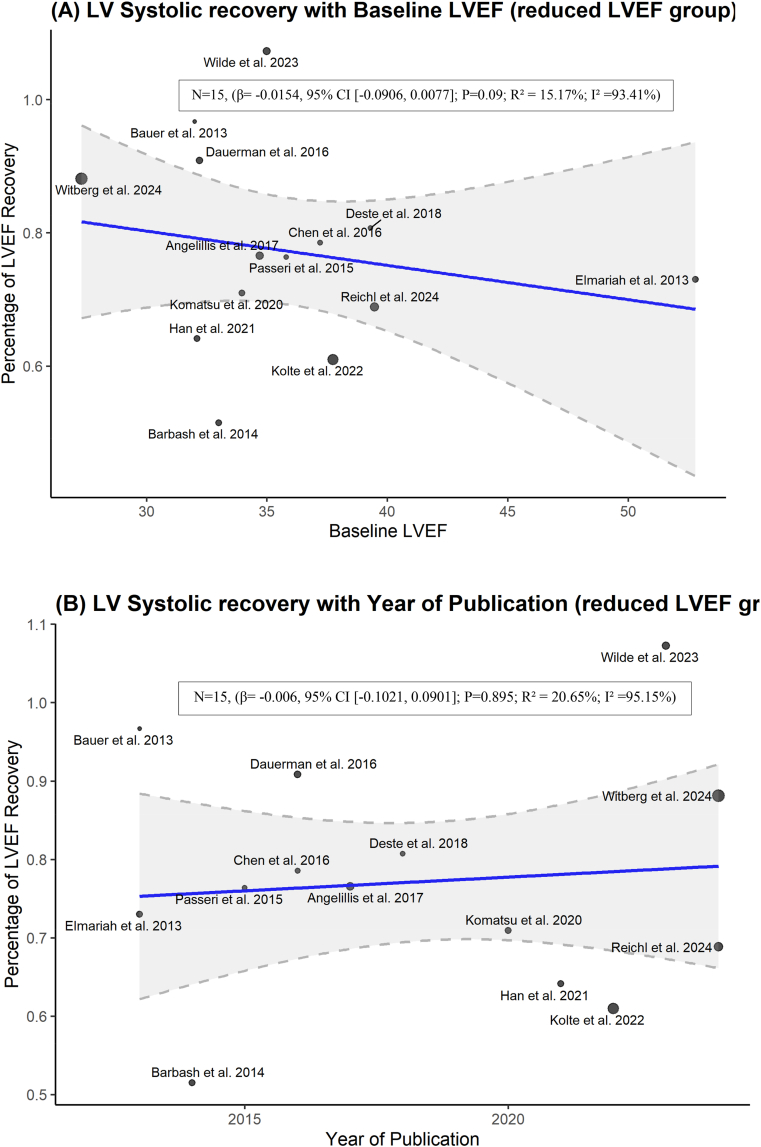
Meta-regression of LV systolic recovery (reduced baseline LVEF subgroup with only ≥10% threshold) against (A) baseline LVEF and (B) publication year.

The meta-regression analysis of all included studies identified that baseline LVEF had a significant negative association with the LV systolic recovery (β = −0.042; 95% CI [−0.075, −0.008]; p = 0.018). However, when excluding the studies that defined recovery with a threshold of ≥5%, the results are no longer significant. Additionally, year of publication was not a significant predictor of the recovery (Fig. 7, Fig. 8).

## 4 Discussion

This systematic review and meta-analysis provides a comprehensive synthesis of the current evidence on LV systolic recovery following TAVI for severe aortic stenosis. The principal findings of this study are threefold: first, TAVI induces a significant improvement in LVEF in a substantial proportion of patients; second, this recovery is most pronounced and frequent in patients with pre-existing LV systolic dysfunction occurring in nearly half of this high-risk population; and third, the strongest and most consistent predictor for the magnitude of this recovery is a lower pre-procedural LVEF, which was found significant in the meta-regression.

### 4.1 Contextualizing the prevalence of LV systolic recovery

This meta-analysis establishes a robust pooled estimate for LV systolic recovery. The finding that 49% of patients with reduced baseline LVEF experience a significant LV recovery, aligns well and consolidates the findings from several studies and registries. For instance, an early analysis of the PARTNER Cohort A trial reported a 51.6% recovery rate (defined as ≥10% LVEF improvement from baseline) in patients with a baseline LVEF <50% [27]. Similarly, the pivotal CoreValve US trial demonstrated a 62.2% systolic recovery rate among patients with a baseline LVEF ≤40% [5,27]. More recently, the large, multicenter AMTRAC registry, which focused on the highest-risk cohort with severe LV dysfunction (LVEF ≤30%), found a recovery rate of 59.5% [20].

The variation in these reported rates, ranging from approximately 33% in the PARTNER 1/2/S3 pooled analysis (mean baseline EF 37.8%) to nearly 60% in cohorts with more severe dysfunction (baseline EF ≤ 30-40%), suggests a coherent biological pattern rather than a contradiction [18,30]. There appears to be a "dose-response" relationship, wherein the myocardium with more severe functional impairment, yet preserved viability, has a greater potential for recovery once the offending afterload is relieved. In the "mixed cohort", the 25% recovery rate in the is a diluted, because a patient with a baseline LVEF of 30% has more capacity to improve by an absolute 10% than a patient with a baseline LVEF of 52%. This leads to a confounded estimate the percentage of LV recovery in this group.

By pooling data from studies with varying severities of "reduced EF" this meta-analysis provides a robust and generalizable central estimate of this phenomenon, anchoring the expected recovery rate at approximately 50% for the broad population of patients with any degree of systolic dysfunction.

### 4.2 Pathophysiological mechanisms and predictors of myocardial recovery

The results of this analysis move beyond simply quantifying recovery to illuminating the underlying pathophysiology. The consistent identification of lower baseline LVEF, higher mean aortic gradient, and the absence of prior myocardial infarction as key predictors points toward a specific myocardial substrate amenable to recovery [5,[18], [19], [20],^24^,^25^,^27^]. This profile describes a ventricle with significant, yet potentially reversible, dysfunction driven by afterload mismatch, a condition often referred to as "myocardial stunning" or "hibernation" on a prolonged timescale. TAVI functions by acutely unloading this viable myocardium, allowing for the restoration of contractile function, reduction in wall stress, and initiation of favorable reverse remodeling.

Conversely, the lack of recovery in many patients, particularly those with a history of MI or extensive coronary artery disease, suggests the presence of irreversible myocardial damage [5,27]. In these cases, contractile units have been permanently lost and replaced by non-contractile scar tissue (replacement fibrosis). In such a ventricle, relieving the afterload cannot restore function to tissue that is no longer viable. This underscores the impact of myocardial fibrosis on LV systolic recovery following TAVI.

The nature and extent of myocardial fibrosis (replacement or interstitial) are critical determinants of patient outcomes and the potential for LV reverse remodeling following intervention. Beyond its mere presence, the distribution of fibrosis, whether focal replacement or diffuse interstitial, appears to hold significant prognostic value. Histological analysis by Puls et al., 2020 [31] in severe aortic stenosis patients revealed a predominance of interstitial fibrosis (72%) over replacement fibrosis (9%). Crucially, their work established that any myocardial fibrosis predicted all-cause mortality, while levels of myocardial fibrosis above the median specifically predicted cardiovascular mortality, where it was more commonly in the highest tertile of myocardial fibrosis (30.3%) than moderate (9.4%), or lowest tertiles (2.9%). Moreover, it was associated with delayed LV reverse remodeling [31].

This underscores the necessity of accurate, non-invasive pre-procedural assessment of myocardial fibrosis. Cardiovascular magnetic resonance (CMR) is the current gold standard, utilizing late gadolinium enhancement (LGE) to identify replacement fibrosis, which was used in patients with aortic stenosis following TAVI [32,33]. While extracellular volume (ECV) quantification to assess interstitial fibrosis, which itself is a powerful predictor of mortality in aortic stenosis [34]. However, in patients with CMR contraindications, cardiac computed tomography (CCT) has emerged as a robust alternative. Supporting this, a meta-analysis of 10 studies [8] found that low pre-procedural ECV, quantified by CCT angiography, was significantly associated with LV systolic recovery post-TAVI(34). Therefore, quantifying the myocardial fibrotic burden, whether by CMR or CCT, provides invaluable data for risk stratification and predicting the trajectory of LV recovery in this high-risk population.

This frames LV systolic recovery not as a stochastic event, but as a predictable consequence of the underlying myocardial tissue characteristics. The results strongly suggest that the benefit of TAVI on LV function is maximized in patients whose cardiomyopathy is primarily valvular in origin, rather than ischemic or idiopathic.

### 4.3 The profound prognostic significance of LV systolic recovery

The most critical implication of this work lies in connecting the primary outcome of LVEF improvement to its profound prognostic significance. The finding that nearly half of patients with baseline dysfunction experience functional recovery is not merely an interesting echocardiographic observation; it represents the proportion of high-risk patients who are shifted onto a trajectory of significantly improved long-term survival. An extensive body of literature has firmly established this link. In Partner-2, the two-year outcomes, all-cause mortality and cardiovascular mortality, and heart failure hospitalization were higher in the group of reduced EF. Even the analysis of the PARTNER trials by Kolte et al. (2022) which demonstrated early LV systolic recovery within 30 days was independently associated with significantly lower 5-year all-cause mortality and cardiac mortality [18,30].

Even more striking are the findings from the AMTRAC registry, which reported that patients with severe baseline dysfunction (LVEF ≤30%) whose LVEF normalized to ≥50% post-TAVI had a lower 3-year mortality risk than patients who started the procedure with a normal LVEF [20]. This remarkable finding suggests that when the LV dysfunction is truly and completely reversible, the prognosis can be excellent. Therefore, the 49% recovery rate identified in this meta-analysis is a powerful surrogate for long-term clinical benefit. It quantifies the substantial portion of patients in whom TAVI does more than just fix a valve, but also it fundamentally alters the course of their underlying heart failure.

### 4.4 Clinical implications and future directions

The findings of this meta-analysis have direct clinical utility. They provide a quantitative, evidence-based foundation for patient counseling and shared decision-making. A patient with severe AS and reduced LVEF, but without extensive coronary artery disease or a history of major MI, can be informed that they have approximately a one-in-two chance of achieving a significant improvement in heart function post-TAVI, an outcome strongly associated with better long-term survival. This can help manage expectations and frame the potential benefits of the procedure.

Following TAVI, some patients may require permanent pacemaker implantation due to post-procedural conduction disturbances, which can impair or delay LV systolic recovery. Pacing-induced cardiomyopathy (PICM) can complicate and delay recovery by compromising left ventricular function and increasing the risk of adverse long-term outcomes. Hayıroğlu et al. demonstrated that pre-implantation Left Ventricular Mass Index (LVMI) is a strong independent predictor of PICM [35]. Furthermore, the recovery trajectory is particularly precarious for elderly patients who develop subsequent arrhythmias. Moreover, the occurrence of atrial fibrillation in octogenarians with dual-chamber pacemakers is a significant independent risk factor for long-term mortality [36]. This highlights that in older populations, the onset of AF following implantation not only hinders functional cardiac recovery but is also a critical prognostic marker for survival.

The new 2025 ESC Guidelines for valvular heart disease endorse intervention for severe aortic stenosis, irrespective of patient symptoms, LVEF, or flow reserve [37]. This guideline-level shift is substantially supported by the findings of this meta-analysis. By providing a robust, quantitative estimate of 49% LV systolic recovery in the reduced LVEF cohort, our work provides key evidence for repositioning TAVI as a restorative, rather than palliative, therapy for patients with aortic stenosis and reduced baseline EF.

For future research, there is a clear need to move beyond clinical predictors toward more precise, imaging-based determinants of myocardial viability. Prospective studies utilizing advanced imaging modalities, such as cardiac CT with myocardial analysis to quantify the burden of myocardial fibrosis, could offer a much more accurate prediction of which patients will experience LV systolic recovery [19,8]. Such tools could refine patient selection for TAVI and other interventions. Furthermore, the field would benefit immensely from the standardization of definitions for LV systolic recovery and the implementation of standardized imaging follow-up protocols in future TAVI trials and registries. This would reduce the heterogeneity that plagues current meta-analysis and allow for more precise and robust conclusions in the future.

Additionally, a study by Cicek et al. demonstrates the superior prognostic accuracy of artificial intelligence (AI) over traditional clinical risk scores, offering a compelling framework for modernizing risk stratification in TAVI [38]. In their development of the "CLASHED" model (incorporating Creatinine, Lymphocyte count, Aortic regurgitation, Stroke history, Hemoglobin, Ejection fraction, and D-dimer), the authors found this model outperform the conventional Revised Cardiac Risk Index (RCRI) in predicting myocardial injury after non-elective surgery (AUC 0.788 vs. 0.611) [38]. Furthermore, their multimodal deep learning (mmDL) model for acute pulmonary embolism utilized CT imaging combined with clinical data to achieve near-perfect mortality prediction (AUC 0.98), far surpassing the standard Pulmonary Embolism Severity Index (PESI, AUC 0.86) [39]. These examples illustrate how AI deep learning models can capture risk more precisely than static additive scores. Adapting this "CLASHED" or “mmDL” models to TAVI could allow for dynamic risk models that incorporate pre-procedural CT scans and frailty markers to outperform current standards like the STS or EuroSCORE II.

### 4.5 Strengths and limitations

This study has several strengths, including: its comprehensive systematic search strategy, its large pooled sample size of over 4700 patients, and the specific focus on synthesizing data for the clinically challenging reduced EF population. The use of pre-specified subgroups and sensitivity analyses adds robustness to the findings.

However, several limitations must be acknowledged. The most significant is the high degree of statistical heterogeneity (I^2^ = 97%) observed in the primary analysis. This is almost certainly a reflection of the substantial clinical and methodological heterogeneity inherent in the included real-world evidence. Key sources of this diversity and heterogeneity is the inclusion of few studies with mixed patient cohorts (combining those with reduced and preserved baseline LVEF) [1,17,19,25]; wide variations in follow-up imaging schedules, including one study (Bauer et al., 2013, n = 31) that reported the LV systolic recovery at 7 days post-TAVI [6], a time point likely too early to reflect true recovery patterns; the application of different thresholds to define LV systolic recovery (≥10% vs. ≥5% LVEF increase from baseline); the use of self-expanding and balloon-expandable valves, and the evolution of TAVI technologies over the 11-year publication span of the included studies. Moreover, the inability to exclude patients with ischemic cardiomyopathy or cardiac amyloidosis in study-level data is a limitation that affects the percentage of patients achieving LV systolic recovery.

This meta-analysis is also based on study-level aggregated data rather than individual patient-level data, which precludes more sophisticated multivariable adjustments to explore the interplay of predictors more deeply. Finally, the included studies were predominantly from high-income countries in North America and Europe, which may limit the generalizability of the findings to other global regions.

## 5 Conclusion

TAVI leads to LV systolic recovery and improvement in nearly half of the patients following the procedure, an effect that is significantly more pronounced in patients with reduced EF at baseline. While the magnitude of improvement varied across studies, nearly half of patients with reduced EF experienced a clinically meaningful increase in LVEF following the procedure.

Several factors, including low baseline LVEF, and absence of hypertension, coronary artery disease or prior percutaneous coronary intervention, were identified as predictors of LV systolic recovery. Recognizing these predictors can help clinicians identify patients who are more likely to benefit from LV systolic recovery and tailor their management strategies accordingly.

Given the well-established link between improved LVEF and better patient outcomes, optimizing TAVI strategies to enhance LV systolic recovery in this high-risk patient population should be a key clinical objective. Future research should focus on identifying novel strategies to further enhance LV systolic recovery and improve long-term outcomes for patients with reduced EF undergoing TAVI.

## CRediT authorship contribution statement

**Elfatih A. Hasabo:** Conceptualization, Data curation, Formal analysis, Investigation, Methodology, Project administration, Resources, Software, Visualization, Writing – original draft, Writing – review & editing. **Ammar Elgadi:** Data curation, Writing – original draft, Writing – review & editing. **Alaa S. Ahmed:** Data curation, Validation, Writing – review & editing. **Esraa S.A. Alfadul:** Data curation, Visualization, Writing – review & editing. **Lina Hemmeda:** Data curation, Writing – review & editing. **Ruman K. Qasba:** Data curation, Writing – review & editing. **Walaa Elnaiem:** Data curation, Writing – review & editing. **Rakhtan K. Qasba:** Data curation, Writing – review & editing. **Hesham Elzomor:** Data curation, Writing – review & editing. **Ratibah Sawabi:** Data curation, Writing – review & editing. **Omar Baqal:** Data curation, Writing – review & editing. **Andreas Rück:** Data curation, Writing – review & editing. **Nawzad Saleh:** Data curation, Writing – review & editing. **Osama Soliman:** Methodology, Supervision, Writing – review & editing.

## Consent to participate

Not applicable.

## Availability of data and materials

Not applicable.

## Ethics approval

This article does not contain any studies with human participants or animals performed by any of the authors.

## Consent for publication

Not applicable.

## Funding

Not applicable.
