# Supplementary material for: Left ventricular systolic recovery after TAVI in severe aortic stenosis: A systematic review and meta-analysis
Source: Int J Cardiol Cardiovasc Risk Prev. 2026 Feb 26;29:200610. doi: 10.1016/j.ijcrp.2026.200610 (PMC12969045; doi:10.1016/j.ijcrp.2026.200610)
Supplement: Multimedia component 1 [file mmc1.docx]

***Supplementary files:***

**Supplementary Table 1**: Search strategy of the studies in databases.

**Supplementary Table 2:** Quality assessment of included studies using adapted NOS

**Supplementary Table 3:** Quality assessment of included studies using Cochrane risk of bias tool.

**Supplementary Table 4:** Positive associated factors with the LVEF improvement after TAVI procedure.

**Supplementary Table 5**: Associated factors with the LVEF improvement after TAVI procedure.

**Supplementary Table 1:** Search strategy of the studies in databases.

| PubMed | (((((((((Transcatheter Aortic Valve Implantation) OR (TAVI)) OR (Transcatheter aortic valve replacement)) OR (TAVR)) OR (Percutaneous aortic valve replacement)) OR (PAVR)) OR (transcatheter heart valve)) OR (THV))) AND ((((((((((((((((((Recovery) OR (Recovered)) OR (Remodeling)) OR (Change in EF)) OR (Change in Ejection Fraction)) OR (Change in left ventricular ejection function)) OR (Change in Left Ventricular Ejection Fraction)) OR (Left Ventricular Ejection Fraction Improvement))) OR (global longitudinal strain)) OR (GLM)) OR (Left ventricular mass index)) OR (LVMi)) OR (Left ventricular global longitudinal strain)) OR (LV-GLS)) OR (ventricular mass index)) OR (normalization)) OR (normalisation)) | 3237 |
| --- | --- | --- |
| Scopus | ( TITLE-ABS-KEY ( ( "transcatheter aortic valve implantation" OR tavi OR "transcatheter aortic valve replacement" OR tavr OR "percutaneous aortic valve replacement" OR pavr OR "transcatheter heart valve" OR thv ) ) AND TITLE-ABS-KEY ( ( recovery OR recovered OR remodeling OR "change in ef" OR "change in ejection fraction" OR "change in left ventricular ejection function" OR "change in left ventricular ejection fraction" OR "left ventricular ejection fraction improvement" OR "global longitudinal strain" OR glm OR "left ventricular mass index" OR lvmi OR "left ventricular global longitudinal strain" OR lv-gls OR "ventricular mass index" OR normalization OR normalisation ) ) ) | 1755 |
| Web of Science | (“Transcatheter Aortic Valve Implantation” OR TAVI OR “Transcatheter aortic valve replacement” OR TAVR OR “Percutaneous aortic valve replacement” OR PAVR OR “transcatheter heart valve” OR THV) (All Fields) and (Recovery OR Recovered OR Remodeling OR “Change in EF” OR “Change in Ejection Fraction” OR “Change in left ventricular ejection function” OR “Change in Left Ventricular Ejection Fraction” OR “Left Ventricular Ejection Fraction Improvement” OR “global longitudinal strain” OR GLM OR “Left ventricular mass index” OR LVMi OR “Left ventricular global longitudinal strain” OR LV-GLS OR “ventricular mass index” OR normalization OR normalisation) (All Fields) | 1071 |
| Cochrane Library | (“Transcatheter Aortic Valve Implantation” OR TAVI OR “Transcatheter aortic valve replacement” OR TAVR OR “Percutaneous aortic valve replacement” OR PAVR OR “transcatheter heart valve” OR THV) AND (Recovery OR Recovered OR Remodeling OR “Change in EF” OR “Change in Ejection Fraction” OR “Change in left ventricular ejection function” OR “Change in Left Ventricular Ejection Fraction” OR “Left Ventricular Ejection Fraction Improvement” OR “global longitudinal strain” OR GLM OR “Left ventricular mass index” OR LVMi OR “Left ventricular global longitudinal strain” OR LV-GLS OR “ventricular mass index” OR normalization OR normalisation) | 192 |
| **Total** |  | 6255 |

**Supplementary Table 2:** Quality assessment of included studies using adapted NOS

| **Study** | **Selection** | **Outcome** | **Total score (maximum is 6)** |
| --- | --- | --- | --- |
| Bauer et al. 2013 (22) | 3 | 2 | 5 |
| Barbash et al. 2014 (16) | 3 | 3 | 6 |
| Passeri et al. 2015 (2) | 3 | 3 | 6 |
| Chen et al. 2016 (23) | 3 | 3 | 6 |
| Eidet et al. 2016 | 3 | 2 | 5 |
| D'Onofrio et al. 2017 (17) | 3 | 3 | 6 |
| Angelillis et al. 2017 (24) | 3 | 3 | 6 |
| Deste et al. 2018 (7) | 3 | 2 | 5 |
| Komatsu et al. 2020 (25) | 3 | 2 | 5 |
| Han et al. 2021 (8) | 3 | 3 | 6 |
| Jeong et al. 2021(1) | 3 | 3 | 6 |
| Kuneman et al. 2022 (26) | 3 | 3 | 6 |
| Kolte et al. 2022 (18) | 3 | 3 | 6 |
| Wilde et al. 2023 (27) | 3 | 3 | 6 |
| Bernhard et al. 2024 (19) | 3 | 3 | 6 |
| Witberg et al. 2024 (20) | 3 | 3 | 6 |

**Supplementary Table 3:** Quality assessment of included studies using Cochrane risk of bias tool.

| **Name** | **Title** | **The Cochrane Collaboration’s tool for assessing risk of bias** | | | | | | | | | | | | | |
| --- | --- | --- | --- | --- | --- | --- | --- | --- | --- | --- | --- | --- | --- | --- | --- |
|  |  | **Random sequence generation (selection bias)** | | **Allocation concealment (selection bias)** | | **Blinding of participants and personnel (performance bias)** | | **Blinding of outcome assessment (Detection bias)** | | **Incomplete outcome data (attrition bias)** | | **Selective reporting (reporting bias)** | | **Other Bias** | |
|  |  | **Low \ High \ Unclear risk of bias** | **Reason** | **Low \ High \ Unclear risk of bias** | **Reason** | **Low \ High \ Unclear risk of bias** | **Reason** | **Low \ High \ Unclear risk of bias** | **Reason** | **Low \ High \ Unclear risk of bias** | **Reason** | **Low \ High \ Unclear risk of bias** | **Reason** | **Low \ High \ Unclear risk of bias** | **Reason** |
|  |  |  |  |  |  |  |  |  |  |  |  |  |  |  |  |
| Dauerman et al. 2016 (5) | Early Recovery of Left Ventricular Systolic Function AfterCoreValve Transcatheter Aortic Valve Replacement | unclear | Reported as "randomized", no clarification of randomization method. | unclear | not stated | high | open-label | high | open-label | low | ITT analysis | low | no selective reporting | low | no |
| Elmariah et al. 2013 (28) | Outcomes of Transcatheter and Surgical Aortic Valve Replacement in High-Risk Patients With Aortic Stenosis and Left Ventricular Dysfunction Results From the Placement of Aortic Transcatheter Valves (PARTNER) Trial (Cohort A) | unclear | Reported as "randomized", no clarification of randomization method. | unclear | not stated | high | open-label | high | open-label | low | minimal loss of follow-up | low | no c reporting | low | no |

# **Supplementary Table 4:** Baseline characteristics stratified by LV systolic recovery status post-TAVI.

| **Study** | **Groups** | **Numbers** | **Age** | **Male, %** | **Logistic EuroSCORE,** | **STS score** | **Baseline EF** | **eGFR (ml/min)** | **Risk factors, number (%)** | | | | | | | | | **NYHA class, number (%)** | | | |
| --- | --- | --- | --- | --- | --- | --- | --- | --- | --- | --- | --- | --- | --- | --- | --- | --- | --- | --- | --- | --- | --- |
|  |  |  |  |  |  |  |  |  | **Hypertension** | **Hypercholesterolemia** | **Diabetes mellitus** | **Smoker** | **Coronary artery diseases** | **Previous** **myocardial infarction** | **Previous revascularization** | **PCI** | **CABG** | **1** | **2** | **3** | **4** |
| Barbash et al. 2014 (16) | Recovered | 24 | 84 ± 8 | 11 (46%) | 32 ± 27 | 11 ± 4 | 30 ± 9 | - | 21 (88%) | - | 5 (21%) | 6 (30%) | 14 (93%) | 8 (33%) | - | 8 (33%) | 8 (33%) | - | - | - | - |
|  | Non-recovered | 75 | 83 ± 6 | 52 (69%) | 39 ± 28 | 12 ± 6 | 34 ± 8 | - | 71 (95%) | - | 29 (39%) | 13 (30%) | 55 (86%) | 20 (29%) | - | 25 (35%) | 41 (56%) | - | - | - | - |
| Dauerman et al. 2016 (5) | Recovered | 97 | 83.0 ± 9.0 | 65 (67%) | - | - | 31.8 ± 7.5 | - | 83 (85.6%) | 84 (86.6%) | 37 (38.1%) | 54 (55.7%) | 86 (88.7%) | 43 (44.3%) | - | 40 (41.2%) | 52 (53.6%) | - | - | - | - |
|  | Non-recovered | 59 | 82.0 ± 9.2 | 44 (74.6%) | - | - | 32.9 ± 6.2 | - | 56 (94.9%) | 50 (84.7%) | 29 (49.2%) | 39 (66.1%) | 53 (89.8%) | 35 (59.3%) | - | 23 (39%) | 34 (57.6%) | - | - | - | - |
| Chen et al. 2016 (22) | Recovered | 30 | 81.5 ± 8.0 | 18 (60.0%) | - | 11.6 ± 5.6 | 35.2 ± 6.5 | - | - | - | - | - | 24 (80%) | 11 (36.7%) | - | 7 (23.3%) | 11 (36.7%) | 0 (0%) | 0 (0%) | 9 (30%) | 21 (70%) |
|  | Non-recovered | 30 | 81.5 ± 6.0 | 21 (70.0%) | - | 11.8 ± 4.3 | 39.2 ± 5.9 | - | - | - | - | - | 27 (90%) | 13 (43.3%) | - | 13 (43.3%) | 17 (56.7%) | 0 (0%) | 0 (0%) | 14 (46.7%) | 16 (53.3%) |
| D'Onofrio et al. 2017 (17) | Recovered | 27 | 79.6 ± 6 | 12 (44.4%) | 21.6 ± 14.3 | 7.3 ± 6.7 | 48.3 ± 10.8 | - | 26 (96.3%) | - | 7 (25.9%) | - | - | 6 (22.2%) | - | - | - | - | - | - | - |
|  | Unchanged | 69 | 80.6 ± 6.4 | 39 (56.5%) | 21.6 ± 11.5 | 8.9 ± 8.2 | 54 ± 11.6 | - | 64 (92.8%) | - | 23 (33.3%) | - | - | 16 (23.2%) | - | - | - | - | - | - | - |
|  | Worsened | 26 | 79.4 ± 5.8 | 10 (38.5%) | 18.6 ± 11.6 | 7.1 ± 8.1 | 62.2 ± 10.2 | - | 24 (92.3%) | - | 5 (19.2%) | - | - | 9 (34.7%) | - | - | - | - | - | - | - |
| Angelillis et al. 2017 (23) | Recovered | 121 | 80.6 ± 7.9 | 64 (52.9%) | 22.7 (14–38) | 7.4 (4.7–12.5) | 33.0 ± 7.7 | - | 96 (79.3%) | - | 28 (23.0%) | - | 29 (24.0%) | - | - | 18 (15.0%) | - |  |  | 99 (81.8%) | |
|  | Non-recovered | 131 | 81.2 ± 8.4 | 66 (50.4%) | 23.1 (17–34) | 5.6 (3.6–8.6) | 36.2 ± 6.9 | - | 101 (77.1%) | - | 37 (28.2%) | - | 43 (33.0%) | - | - | 39 (29.8%) | - |  |  | 100 (76.3%) | |
| Komatsu et al. 2020 (24) | Recovered | 42 | 82.0± 8.6 | 29 | - | 8.6 (5.3–11.3) | 27.5 (20.0–40.0) |  | 37 (88.1%) | - | 17 (40.5%) | - | - | 15 (35.7%) | - | 12 (28.6%) | 11 (26.8%) | - | - | - | - |
|  | Non-recovered | 57 | 81.4 ± 9.5 | 44 | - | 8.0 (4.7–10.7) | 38.0 (31.5–43.0) | - | 52 (91.2%) | - | 20 (35.1%) | - | - | 25 (43.8%) | - | 21 (37.5%) | 13 (23.6%) | - | - | - | - |
| Han et al. 2021(8) | Recovered | 39 | 81.3 ± 9.6 | 25 (64.1%) | - | - | 30.6 ± 10.8 | - | 30 (76.9%) | - | 15 (38.5%) | - | - | 4 (10.3%) | 9 (23.1%) | 4 (10.3%) | 6 (15.4%) | - | - | - | - |
|  | Non-recovered | 70 | 79.3 ± 9.8 | 58 (82.9%) | - | - | 32.9 ± 9.7 | - | 48 (68.6%) | - | 24 (34.3%) | - | - | 16 (22.9%) | 30 (42.9%) | 17 (24.3%) | 19 (27.1%) | - | - | - | - |
| Jeong et al. 2021 (1) | Recovered | 160 | 79.9 ± 5.1 | 86 (53.8%) | 14.2 ± 11.1 | 3.8 ± 2.4 | - | - | 130 (81.2%) | 115 (71.9%) | 71 (44.4%) | - | - | 6 (3.8%) | - | 43 (26.9%) | 7 (4.4%) | - | - | - | - |
|  | Non-recovered | 534 | 79.7 ± 5.5 | 267 (49.4%) | 12.2 ± 10.9 | 4.0 ± 3.0 | - | - | 480 (88.9%) | 412 (76.3%) | 275 (50.9%) | - | - | 25 (4.6%) | - | 153 (28.3%) | 26 (4.8%) | - | - | - | - |
| Kolte et al. 2022 (18) | Recovered | 216 | 83.2 ± 7.4 | 129 (59.7%) | - | 8.9 ± 4.4 | 35.6 ± 9.1 | - | 189 (87.5%) | - | 71 (32.9%) | - | 162 (75.0%) | 46 (21.3%) |  | 73(33.8%) | 61 (28.2%) |  |  | 197 (91.2%) | |
|  | Non-recovered | 443 | 82.1 ± 7.9 | 339 (76.5%) | - | 8.1 ± 4.4 | 38.8 ± 8.4 | - | 409 (92.3%) | - | 174(39.3%) | - | 367(82.8%) | 154(34.8%) |  | 163(36.9%) | 190 (42.9%) |  |  | 382 (86.2%) | |
| Bayramoğlu et al. 2023 (29) | Recovered | 18 | 74.8 ± 5.3 | 4 (22.2%) | 13.9 ± 2.4 | - | - | - | 13 (72.2%) | - | 4 (22.2%) | 4 (22.2%) | 5 (27.8%) | - | - | - | - | - | - | - | - |
|  | Non-recovered | 42 | 78.3 ± 5.0 | 23 (54.8%) | 14.1 ± 2.2 | - | - | - | 34 (81.0%) | - | 29 (69%) | 9 (21.6%) | 21 (50%) | - | - | - | - | - | - | - | - |
| Wilde et al. 2023 (26) | Recovered | 169 | 80 ± 6 | 103 (60.9%) | 33.6 ± 16.9 | 8.1 ± 6.2 | 33.0 ± 9.9 | 49.4 ± 21.6 | - | - | 52 (30.8%) |  | 124 (73.4%) | 52 (30.8%) | - | 73 (43.2%) | - | - | - | - | 34 (20.1%) |
|  | Non-recovered | 50 | 80 ± 6 | 38 (76%) | 34.4 ± 19.9 | 7.6 ± 6.7 | 40.1 ± 7.5 | 49.1 ± 17.5 | - | - | 18 (36%) |  | 43 (86%) | 11 (22%) | - | 30 (60%) | - | - | - | - | 6 (12%) |
| Witberg et al. 2024 (20) | No EF recovery | 370 | 78.9±7.9 | 264 (71.4%) | - | 7.0±1.6 | 26.4±2.1 | 51.3±25.1 | 284 (76.7%) | - | 133 (35.9%) | - | - | 162 (43.8%) | - | 169 (45.6%) | 83 (22.5%) | - | - | 311 (84.1%) | |
|  | EF recovery | 300 | 80.2±7.1 | 202 (67.4%) | - | 7.1±1.8 | 27.0±1.9 | 55.1±25.5 | 229 (76.2%) | - | 111 (37.0%) | - | - | 67 (22.3%) | - | 101 (33.7%) | 57 (19.2%) | - | - | 250 (83.3%) | |
|  | EF normalisation | 244 | 80.0±8.2 | 137 (55.9%) | - | 7.9±1.9 | 28.1±1.1 | 54.0±27.1 | 189 (77.4%) | - | 84 (34.3%) | - | - | 36 (14.7%) | - | 53 (21.7%) | 23 (9.6%) | - | - | 191 (78.3%) | |
| Data are presented as number (percentage), mean ± SD, median (IQR)  Abbreviation: CABG: coronary artery bypass grafting, eGFR: estimated glomerular filtration rate, IQR: Interquartile range; NYHA: New York Heart Association, PCI : Percutaneous coronary intervention, STS: Society of Thoracic Surgery | | | | | | | | | | | | | | | | | | | | | |

**Supplementary Table 5**: Associated factors with the LVEF improvement after TAVI procedure.

| **Study** | **Univariate** | | | | **Multivariate** | | | |
| --- | --- | --- | --- | --- | --- | --- | --- | --- |
|  | **Variables** | **OR** | **CI** | **P value** | **Variables** | **OR** | **CI** | **P value** |
| Kuneman et al. 2022 (26) | Hypertension | 1.555 | 1.068-2.265 | 0.021 | CAD | 1.796 | 1.055-3.057 | 0.031 |
|  | CAD | 1.776 | 1.254-2.515 | 0.001 | Previous MI | 2.072 | 1.189-3.611 | 0.01 |
|  | Previous myocardial infarction | 1.833 | 1.176-2.858 | 0.007 | Baseline LEFV <50% | 0.224 | 0.145-0.346 | <0.001 |
|  | PCI | 1.578 | 1.087-2.291 | 0.016 | Permanent pacemaker | 1.931 | 1.245-2.995 | 0.003 |
|  | Atrial fibrillation | 0.618 | 0.423-0.902 | 0.013 | - | - | - | - |
|  | Baseline LEFV <50% | 0.257 | 0.179-0.368 | <0.001 | - | - | - | - |
|  | Permanent pacemaker | 1.548 | 1.066-2.249 | 0.022 | - | - | - | - |
| Dauerman et al. 2016 (5) | Baseline mean gradient >40 mmHg | 3.28 | 1.59–6.75 | 0.001 | Baseline mean gradient >40 mmHg | 4.59 | 1.76–11.96 | 0.002 |
|  | Baseline peak aortic valve velocity | 2.59 | 1.43–4.69 | 0.002 | - | - | - | - |
| Chen et al. 2016 (23) | Higher basline heart rate | - | - | 0.02 | - | - | - | - |
|  | Low baseline LVEF % | - | - | 0.01 | - | - | - | - |
| Elmariah et al. 2013 (28) | Previous MI | 0.53 | 0.28, 1.00 | 0.048 | Permanent pacemaker | 0.34 | 0.15 -0.77 | 0.01 |
|  | Permanent pacemaker | 0.41 | 0.20, 0.83 | 0.014 | Baseline LVEF | 0.91 | 0.86- 0.95 | <0.0001 |
|  | Baseline LVEF | 0.93 | 0.89 - 0.97 | 0.0004 | Mean AVG | 1.03 | 1.01-1.06 | 0.03 |
| Kolte et al. 2022 (18) | Diabetes | 0.55 | 0.34-0.86 | 0.01 | BMI | 1.06 | 1.02-1.10 | <.001 |
|  | Cancer | 0.5 | 0.32-0.80 | 0.003 | Diabete | 0.61 | 0.40-0.92 | 0.02 |
|  | Baseline LVEF, % | 0.93 | 0.90-0.96 | <.001 | Prior MI | 0.65 | 0.42-0.98 | 0.04 |
|  | LVEDD, cm | 0.61 | 0.43-0.87 | 0.006 | Cancer | 0.56 | 0.37-0.86 | 0.008 |
|  | AV area, cm2 | 0.16 | 0.03-0.78 | 0.02 | Baseline LVEF, % | 0.93 | 0.90-0.95 | <.001 |
|  | BMI | 1.06 | 1.02-1.10 | 0.005 | LVEDD, cm | 0.59 | 0.44-0.78 | <.001 |
|  | - | - | - | - | AV area, cm2 | 0.19 | 0.05-0.73 | 0.02 |
|  | - | - | - | - | LVOT Doppler stroke volume inde | 1.03 | 1.00-1.06 | 0.04 |
| Komatsu et al. 2020 (25) | ZVa>5 mmHg/ml/m2 | 3.88 | 1.58–9.99 | 0.0028 | ZVa>5 mmHg/ml/m2 | 3.31 | 1.05–11.8 | 0.045 |
|  | EF<30% | 3.08 | 1.33–7.31 | 0.008 |  |  |  |  |
| Wilde et al. 2023 (27) | LVEF at baseline (%) | 0.92 | 1.07-1.96 | <0.01 | Stroke volume index <25 mL/m2 | 2.31 | 1.08-3.58 | <0.01 |
|  | LVEF <30% | 1.9 | 1.12-2.01 | <0.01 | LVEF at baseline (%) | 0.8 | 0.98-1.73 | <0.01 |
|  | - | - | - | - | LVEF <30% | 2.76 | 1.53-2.91 | <0.01 |
|  | - | - | - | - | TR moderate or more | 1.96 | 1.14-3.72 | 0.02 |
|  | Hypertension | 0.54 | 0.34−0.87 | 0.01 | Hypertension | 0.56 | 0.34−0.94 | 0.03 |
|  | Previous heart failure | 1.76 | 1.13−2.72 | 0.01 | Baseline significant ARz | 0.48 | 0.27−0.85 | 0.01 |
| Jeong et al. 2021(1) | Pre-dilatation before valve implantation | 1.64 | 1.08−2.50 | 0.02 | LV mass index (per 100 g/m2) | 1.07 | 1.01−1.13 | 0.02 |
|  | Average diameter of annulus | 1.09 | 1.01−1.18 | 0.03 | - | - | - | - |
|  | LV mass index (per 100 g/m2) | 1.08 | 1.03−1.13 | < 0.001 | - | - | - | - |
|  | Coronary artery disease | 0.33 | 0.12–0.88 | 0.027 | Coronary artery disease | 0.3 | 0.11–0.84 | 0.022 |
|  | Pacer lead | 0.24 | 0.07–0.86 | 0.028 |  |  |  |  |
| Han et al. 2021 (8) | Sex | 0.37 | 0.15–0.91 | 0.031 | AV mean gradient (mmHg) | 1.07 | 1.03–1.11 | <0.001 |
|  | Prior revascularization | 0.40 | 0.17–0.97 | 0.042 | LVEDV (mL) | 0.99 | 0.98–0.99 | 0.035 |
|  | AV mean gradient (mmHg) | 1.07 | 1.03–1.11 | <0.001 | ECV (%) | 0.92 | 0.86–0.99 | 0.018 |
|  | AV peak gradient (mmHg) | 1.02 | 1.00–1.04 | 0.025 | - | - | - | - |
|  | AVA index (cm2/m2) | 0.01 | 0.00–0.57 | 0.024 | - | - | - | - |
|  | LVEDV | 0.99 | 0.98–0.99 | 0.040 | - | - | - | - |
|  | ECV >_30% | 0.43 | 0.19–0.96 | 0.039 | - | - | - | - |
|  | ECV (per %) | 0.92 | 0.87–0.98 | 0.012 | - | - | - | - |
| Bernhard et al. 2024 (19) | LV EDV indexed [ml/m2] | 1.014 | 1.007 - 1.020 | <0.001 |  | - | - | - |
|  | LV Mass indexed [g/m2] | 0.999 | 0.988-1.010 | 0.858 | LV Mass indexed | - | - | - |
|  | LV EF | 0.981 | 0.968 -0.994 | 0.003 | LV EF | 0.972 | 0.945-0.999 | 0.048 |
|  | LV GLS | 1.048 | 1.005-1.094 | 0.029 | LV GLS | 0.916 | 0.838 - 1.002 | 0.056 |
|  | LV GCS | 1.030 | 0.999 - 1.061 | 0.059 | LV GCS | - | - | - |
|  | LV GRS | 0.987 | 0.979 -0.995 | 0.002 | LV GRS | - | - | - |
|  | RV EDA | 1.017 | 0.987 - 1.047 | 0.266 | RV EDA | - | - | - |
|  | RV FAC | 0.982 | 0.964 1.000 | 0.044 | RV FAC | - | - | - |
|  | RV GLS | 1.047 | 1.018- 1.078 | 0.002 | RV GLS | 1.021 | 0.985 - 1.057 | 0.253 |
|  | LA EF | 0.972 | 0.961 - 0.984 | <0.001 | LA EF | 0.982 | 0.968- 0.996 | 0.011 |
|  | LA GLS | 0.955 | 0.931 - 0.979 | <0.001 | LA GLS | - | - | - |
| Witberg et al. 2024 (20) | - | - | - | - | Previous MI | 0.45 | 0.28-0.71 | <0.001 |
|  | - | - | - | - | eGFR <60 mL/min | 0.49 | 0.32-0.77 | 0.002 |
|  | - | - | - | - | Mean AVG (per mmHg) | 1.02 | 1.01-1.04 | 0.007 |
|  | - | - | - | - | LF-LG AS | 0.50 | 0.29-0.84 | 0.009 |
| Abbreviation: CAD: Coronary artery disease, CI: Confidence interval, EDA /EDV: end diastolic area, EF: ejection fraction, FAC: fractional area change, GCS: global circumferential strain, GLS: global longitudinal strain, GRS: global radial strain, LV /RV: left/right ventricle | | | | | | | | |
